# Changes in Structure and Reactivity of Ng_2_ Encapsulated in Fullerenes: A Density Functional Theory Study

**DOI:** 10.3389/fchem.2020.00566

**Published:** 2020-07-03

**Authors:** Meng Li, Xin He, Bin Wang, Dongbo Zhao, Chunying Rong, Pratim K. Chattaraj, Shubin Liu

**Affiliations:** ^1^Department of Chemistry, College of Chemistry and Chemical Engineering, Hunan Normal University, Changsha, China; ^2^School of Chemistry and Chemical Engineering, Nanjing University, Nanjing, China; ^3^Department of Chemistry and Center for Theoretical Studies, Indian Institute of Technology, Kharagpur, India; ^4^Research Computing Centre, University of North Carolina, Chapel Hill, NC, United States

**Keywords:** noble gas dimer, fullerene, density functional theory, information-theoretic approach, encapsulation

## Abstract

Noble gas can be no noble in certain situations from the perspective of structure, bonding, and reactivity. These situations could be extreme experimental conditions or others. In this contribution, we systematically investigate the impact of fullerene encapsulation on molecular structure and chemical reactivity of noble gas dimers (Ng_2_) in a few fullerene molecules. To that end, we consider He_2_, Ne_2_, and Ar_2_ dimers encapsulated in C_50_, C_60_, and C_70_ fullerenes. We unveil that bond distances of Ng_2_ inside fullerene become substantially smaller and noble gas atoms become more electrophilic. In return, these noble gas dimers make fullerene molecules more nucleophilic. Using analytical tools from density functional theory, conceptual density functional theory, and information-theoretic approach, we appreciate the nature and origin of these structure and reactivity changes. The results and conclusions from this work should provide more new insights from the viewpoint of changing the perspectives of noble gas reactivity.

## Introduction

Regarded as the most unreactive elements in the periodic table, noble gas atoms could indeed be reactive and no noble at all under certain circumstances, [e.g., high temperature, high pressure, special conditions (e.g., confinement, etc.)]. The first successful experimental realization was the synthesis of xenon hexafluoro platinate XePtF_6_ in 1962 (Bartlett, 1962; Graham et al., 2000). Since then many more compounds with noble gas elements included have been reported (Turner and Pimentel, 1963; Nelson and Pimentel, 1967; Bondybey, 1971; Stein, 1973; Holloway and Hope, 1999) and a brand new discipline of noble gas chemistry has emerged thereafter.

In this contribution, we explore the possibility of fullerene encapsulation as another feasible way to make noble gas elements no longer noble. In specific, we consider the impact of the encapsulation by fullerene molecules on geometrical structure and chemical reactivity for noble gas species. Previously, by heating fullerenes at 650°C under 3,000 atmosphere, helium (Saunders et al., 1993), neon (Saunders et al., 1993), argon (DiCamillo et al., 1996), krypton (Yamamoto et al., 1999), and xenon (Syamala et al., 2002) noble gas atoms were experimentally introduced into the fullerene cage. This experimental realization of fullerene encapsulation demonstrates the feasibility, and there were reports of experimental realization of noble gas dimers encapsulation in fulerenes (Saunders et al., 1996; Laskin et al., 1998; Peres et al., 2001; Popov et al., 2013; Saha et al., 2019). Also, earlier, Krapp and Frenking performed a computational study on Ng_2_@C_60_ (Ng = He, Ne, Ar, Kr, and Xe) systems from the bonding perspective and concluded that He_2_@C_60_ and Ne_2_@C_60_ were weakly bonded van der Waals complexes (Krapp and Frenking, 2007). On the other hand, Solà et al. explored the same systems from the reactivity perspective and they attributed the changes in reactivity to the stabilized LUMO, increased fullerene strain energy, and compressed Ng_2_ unit (Osuna et al., 2009). There are other theoretical and computational studies on the encapsulation of either noble gas or other species in the literature (Osuna et al., 2011; Cheng and Sheng, 2013; Dolgonos and Peslherbe, 2014; Khatua et al., 2014; Bickelhaupt et al., 2015; Kryachko et al., 2015; Nikolaienko and Kryachko, 2015; Jalife et al., 2016; Srivastava et al., 2016; Nikolaienko et al., 2017; Foroutan-Nejad et al., 2018; Jaroš et al., 2018; Gómez and Restrepo, 2019; Das et al., 2020).

In this work, we reconsider the likelihood of these species from the computational perspective using a few newly developed theoretical tools. To that end, we examine noble gas dimers, He_2_, Ne_2_, and Ar_2_, inside a few fullerene systems, C_50_, C_60_, and C_70_, as illustrative examples in this work. In addition, to pinpoint the nature and origin of these structure and reactivity changes, we make use of a few analytical tools available in the literature, including density functional theory, conceptual density functional theory, information-theoretic approach, energy decomposition analysis, bonding energy analysis, natural population analysis, and noncovalent interaction analysis. In information theory, Shannon introduced a measure of information content or a lack of it as an entropy through an analogy with the statistical mechanical variant of thermodynamic entropy as proposed by Boltzmann. Hence the stability of a system and spontaneous evolution toward an equilibrium state can be understood by following the variation of the entropy during the thermodynamic process. These analyses should provide better understanding from the viewpoint of changing the perspectives of noble gas reactivity.

## Computational Details

### Models

Nine model systems were built to examine the impact of fullerene encapsulation on structure and reactivity properties for noble gas dimers (Ng_2_) using three fullerenes C_50_, C_60_, and C_70_ and three Ng_2_ species, He_2_, Ne_2_, and Ar_2_. They are represented by following notations: He_2_@C_50_, He_2_@C_60_, He_2_@C_70_, Ne_2_@C_50_, Ne_2_@C_60_, Ne_2_@C_70_, Ar_2_@C_50_, Ar_2_@C_60_, and Ar_2_@C_70_. Also, as the reference, the structural and reactivity properties of these three fullerene molecules and three Ng_2_ dimers in vacuum were also simulated and compared.

### Analyses

Structure, bonding and reactivity properties for these species were analyzed through a number of well-established analytical tools available in the literature. They include analyses of the total energy decomposition (Parr and Yang, 1989; Liu, 2007), interaction energy decomposition (Bickelhaupt and Baerends, 2000), natural population (Glendening et al., 2012), non-covalent interactions (Johnson et al., 2010), conceptual density functional theory (Geerlings et al., 2003, 2020; Liu, 2009, 2013), and informational-theoretic approach (Liu, 2016; Rong et al., 2019). These analyses enable in-depth understanding about the origin and nature of the changes in structure, bonding, and reactivity. Formulations as well as the details of how to perform these analyses are available elsewhere.

### Methodologies

Structure optimizations and natural population analyses were performed with the Gaussian 16 (Frisch et al., 2016) package (versionA03), with ultrafine integration grids and tight self-consistent field (SCF) convergence. To obtain the most stable orientation of Ng_2_ dimer in a fullerene cage, quantum molecular dynamics simulations were performed for each species at the DFT B3LYP/3-21G (Lee et al., 1988; Becke, 1993) level of theory at 3,000 K for 300 ps with a step size of 1 fs. A total of 20 structures with the lowest total energy were selected as the possible structure candidates from the trajectory and then optimized at the DFT M06-2X/6-311G(d) (Zhao and Truhlar, 2008) level of theory. The structure with the lowest total energy from these 20 candidates was selected as the global minimum for the species. The full structure optimization was followed by a single-point frequency calculation to ensure that the final optimized structure has no imaginary frequency. As a measure of quality control, we conducted a benchmark test on the impact of different choices of basis sets and approximate DFT functionals (Ma et al., 2014). The Multiwfn 3.6.1 program (Lu and Chen, 2012) was utilized to calculate the information-theoretic quantities by using the checkpoint file from the Gaussian calculations as the input file. The total energy components were obtained from Gaussian calculations with the keyword of iop(5/33 = 1). The ADF (Amsterdam Density Functional) (Lee et al., 1988) package was employed to perform the bond energy decomposition (BED) analysis. The BED analysis was conducted using the previously optimized structure and the DFT BP86 approximate functional with the double zeta basis set, zero order regular approximation for relativistic correction, and Grimme4 dispersion correction (Te Velde et al., 2001).

## Results and Discussion

Table 1 is the benchmark results using Ar_2_@C_60_ as an example with 14 basis sets and 14 DFT functionals as well as HF and MP2 methods to examine their impact on the Ar-Ar bond distance. It is compared with Ar-Ar length in vacuum. As can be seen from the table, the bond length in Ar2@C_60_ is not significantly dependent on the choice of basis sets and functionals, which is unsurprising because the Ng_2_ molecule is confined. As a reference, the experimental Ar-Ar length in vacuum is 3.758 Å (Computational Chemistry Comparison Benchmark DataBase, 2019). There are a handful of computational results for the Ar-Ar length (Colburn and Douglas, 1976). In all analyses below, we chose M06-2X/6-311G(d).

**Table 1:** Benchmark tests for Ar_2_@C_60_ with 14 basis sets and 14 functionals/methods to examine the impact of the methodology dependence on the Ar-Ar bond distance in both vacuum and C_60_ fullerene.

**Basis set**	**Vacuum**	**C_**60**_**	**Functional**	**Vacuum**	**C_**60**_**
STO-3G	3.619	2.381	lsda	3.383	2.356
3-21G	3.395	2.388	blyp	5.709	2.376
6-31G(d)	3.971	2.353	b3lyp	6.481	2.359
6-311G(d)	4.039	2.353	pw91pw91	3.835	2.370
6-311G(d,p)	4.039	2.353	cAM-b3lyp	3.823	2.351
6-311+G(d)	4.174	2.352	b3pw91	6.198	2.357
6-311++G(d,p)	4.174	2.352	pbepbe	3.878	2.372
Def2SVPP	3.360	2.352	hseh1pbe	3.905	2.355
Def2TZVP	4.015	2.347	hcth	3.838	2.354
DGDZVP	4.031	2.355	tpsstpss	4.083	2.374
cc-pVDZ	3.741	2.355	ωb97xd	4.147	2.344
cc-pVTZ	4.029	2.349	m06-2x	3.971	2.353
aug-cc-pVTZ	4.041	2.352	hf	4.525	2.338
CBSB7	4.034	2.350	mp2	4.173	2.352

*When basis sets were tested, M06-2X functional was employed. When functionals were tested, 6-31G(d) basis set was employed. Units in Å*.

**Figure 1:**
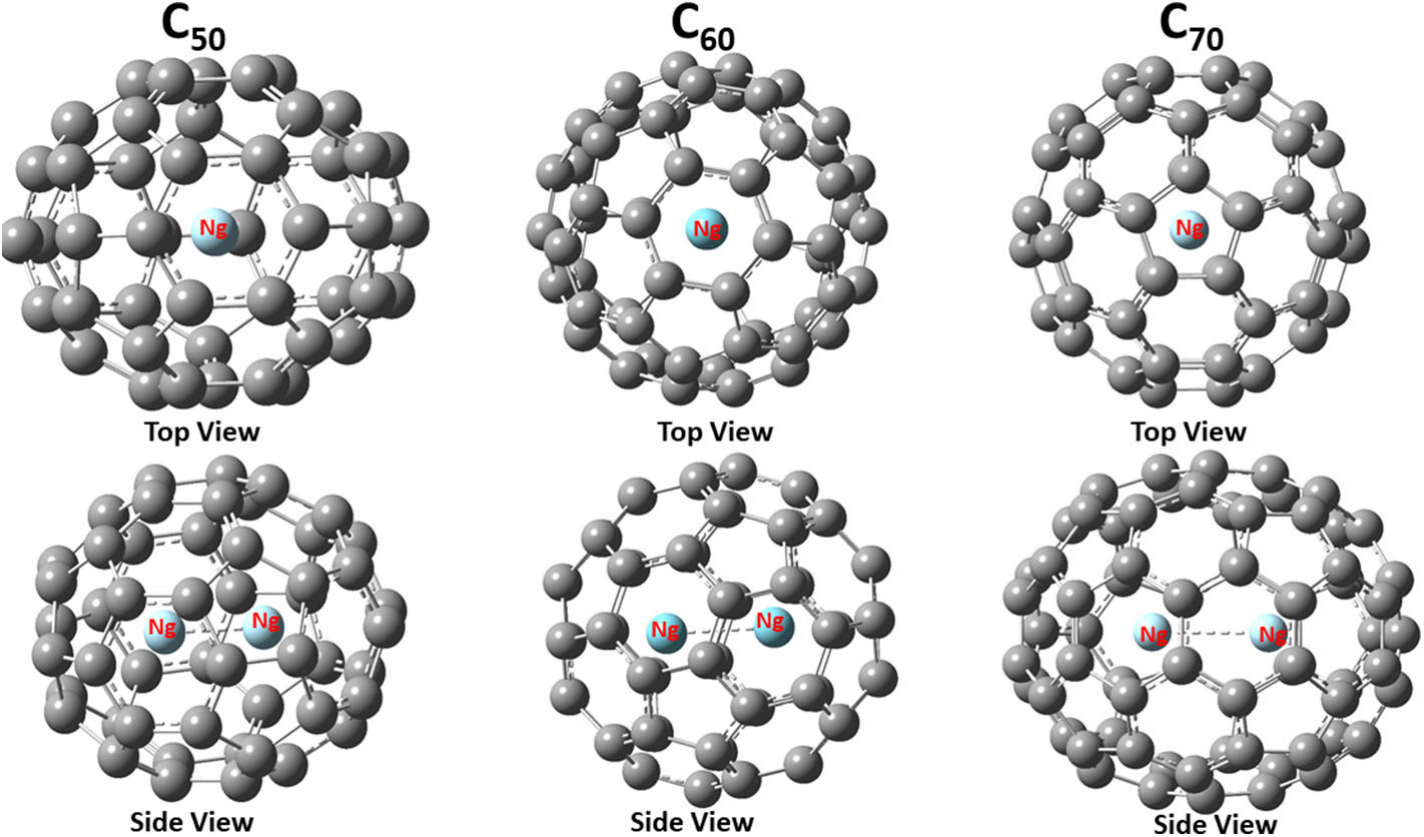
Optimized structures of noble gas dimers encapsulated in fullerene cages with Ng = Ar as an illustrative example. The point group for these three encapsulated systems are C_2v_, D_3d_, and D_5h_, respectively.

Figure 1 shows the final structure obtained for Ng_2_@C_50_, Ng_2_@C_60_, and Ng_2_@C_70_ with both top and side views. With a given fullerene, the binding mode is the same for different Ng_2_ dimers, but for different fullerenes, as shown in Figure 1, the same Ng_2_ dimer prefers to bind differently. This is not surprising either. This is because, for instance, C_70_ is elongated so the dimer aligns with the long axis, as one would expect from a simple steric argument. These similarity and difference illustrate the fact that the size and nature of fullerene molecules play more important role during the encapsulation process of Ng_2_ dimers.

Table 2 exhibits a few selected structural and electronic properties of these species. As can be seen from the table, the bond length of Ng_2_ dimers becomes smaller when encapsulated in fullerenes, and the smaller the fullerene cage the shorter the Ng-Ng distance. This latter trend was resulted from the fact that a smaller fullerene has a smaller cage inside so Ng_2_ dimers are forced to have shorter bond distance. Energetically, both BSSE (basis set superposition error; Boys and Bernardi, 1970; Simon et al., 1996)-corrected and uncorrected interaction energy results show that for He_2_, all three fullerene molecules yield negative (favorable) interaction energies, whereas for larger Ng_2_ dimers, (e.g., Ne_2_ and Ar_2_, it appears that only larger-sized fullerenes such as C_70_ are able to yield attractive interactions between Ng_2_ and fullerene, albeit with the limitations of the method of computation used). This is because larger Ng_2_ dimers tend to occupy more space within the fullerene molecule, making it harder for smaller-sized fullerenes to compensate the energetic cost from electrostatic and other repulsions. We are, however, interested in analyzing the qualitative trends only.

**Table 2:** A few selected structure and electronic properties at the DFT M06-2X/6-311G(d) level of theory for Ng_2_ (Ng = He, Ne, and Ar) dimers encapsulated in C_50_, C_60_, and C_70_ fullerenes^a^.

**Ng-Ng**	**Property**	**Vacuum**	**C_**50**_**	**C_**60**_**	**C_**70**_**
He-He	R (Å)	2.862	1.835	1.984	2.559
	Point group		C_2v_	D_3d_	D_5h_
	${\text{E}}_{\text{int}}^{\text{BSSE}}$ (kcal/mol)		−1.47	−4.36	−6.73
	E_int_ (kcal/mol)		−2.42	−5.13	−7.39
	Hirshfeld charge on Ng		0.073	0.056	0.047
	NPA charge on Ng		0.002	0.002	0.002
Ne-Ne	Distance (Å)	2.685	1.971	2.090	2.557
	Point group		C_2v_	D_3d_	D_5h_
	${\text{E}}_{\text{int}}^{\text{BSSE}}$ (kcal/mol)		15.12	1.31	−8.13
	E_int_ (kcal/mol)		4.71	−7.15	−15.36
	Hirshfeld charge on Ng		0.127	0.091	0.067
	NPA charge on Ng		0.010	0.009	0.008
Ar-Ar	Distance (Å)	4.041	2.235	2.352	2.667
	Point group		C_2v_	D_3d_	D_5h_
	${\text{E}}_{\text{int}}^{\text{BSSE}}$ (kcal/mol)		131.41	53.81	−7.06
	E_int_ (kcal/mol)		126.52	49.91	−10.17
	Hirshfeld charge on Ng		0.180	0.168	0.161
	NPA charge on Ng		−0.047	−0.007	0.003

a *R is the bond distance between Ng atoms; ${\text{E}}_{\text{int}}^{\text{BSSE}}$ and E_int_ are the interaction energy between Ng_2_ dimer and the fullerene molecule with and without the BSSE (basis set superposition error) correction considered, respectively; Hirshfeld is the Hirshfeld charge based on the stockholder partition of atoms in molecules; and NPA charge is the electron charged based on the natural population analysis*.

Shown in Table 2 are two charge results for Ng atoms encapsulated in fullerene molecules. The first one is the NPA (natural population analysis) charge from NBO (natural bond orbital) analysis (Glendening et al., 2012), and the other is the Hirshfeld charge based on the shareholder partition of atoms in molecules (Hirshfeld, 1977). NPA charges for Ng atoms could be either positive or negative, but the Hirshfeld charge value is always positive for Ng atoms. According to the reference literature on the Hirshfeld charge (Liu et al., 2014), which is a good descriptor of electrophilicity and nucleophilicity, a positive Hirshfeld charge is an indication that the atom is electrophilic. The larger value of a positive Hirshfeld charge indicates that it is more electrophilic, able to accept more electrons from a nucleophile. Hirshfeld charge results in Table 2 displays that Ng_2_ in smaller fullerenes tend to be more electrophilic.

**Figure 2:**
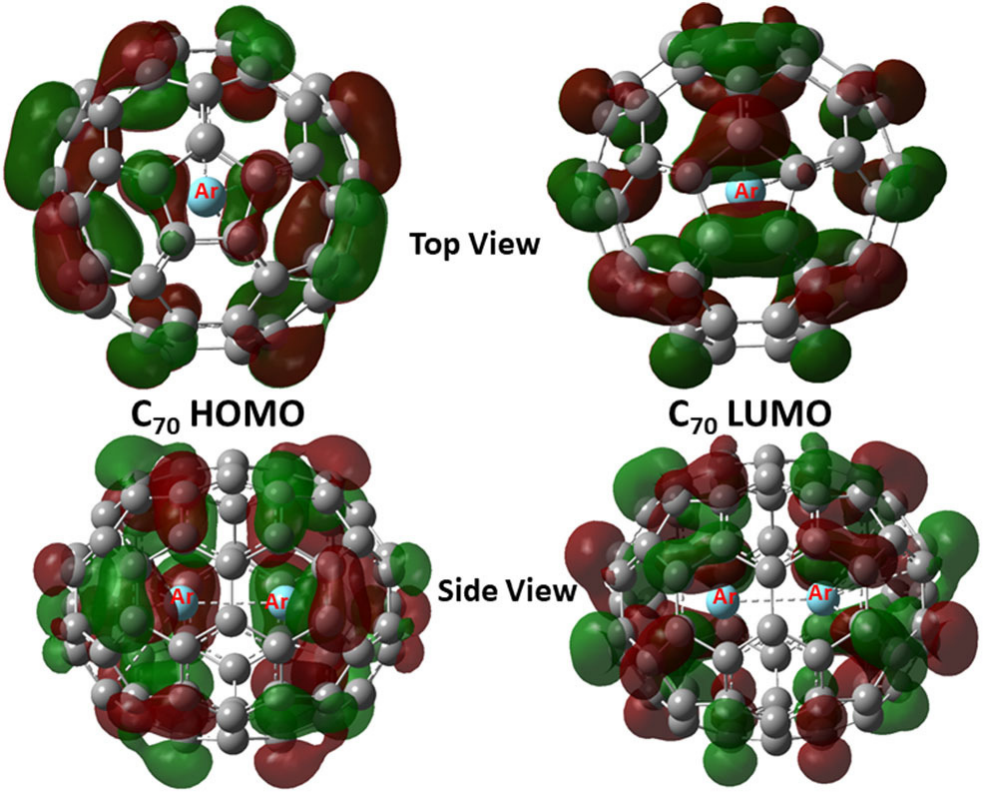
HOMO and LUMO contour surfaces of Ar_2_@C_70_ molecule.

Shown in Figure 2 are frontier molecular orbitals from both top and side views for Ar_2_@C_70_ as an illustrative example, from which one can see that they are both delocalized over the entire molecule. Notice that in both orbitals, we do not see discernible contributions from the Ng_2_ dimer overlapping with any of the atomic orbitals from the fullerene, suggesting that atomic orbitals from Ng atoms are not involved in the frontier molecular orbital delocalization. In Table 3, using the information from these frontier orbitals, we calculated a few global reactivity descriptors from conceptual DFT, including chemical potential μ (Parr et al., 1978), chemical hardness η (Parr and Pearson, 1983), and electrophilicity index ω (Parr et al., 1999) for the nine systems studied in this work. A larger value in hardness is usually an indication of being more stable, while a larger value in electrophilicity index suggests that the species is more reactive. From the results in Table 2, we can see that numerical values of these reactivity indices are slightly changed after Ng_2_ encapsulation. This should be mainly due to the charge transfer between Ng_2_ and fullerene. Ng_2_ in smaller fullerenes are more reactive with a larger ω value, whereas in larger fullerenes it becomes often more stable with a larger η value. These results agree well with the conventional chemical wisdom.

**Table 3:** Global descriptors from Conceptual DFT, including HOMO, LUMO, chemical potential μ, chemical hardness η, and electrophilicity index ω for the systems studied in this work.

**Ng_**2**_**	**Fullerene**	**HOMO**	**LUMO**	**μ**	**η**	**ω**
	C_50_	−0.263	−0.150	−0.206	0.114	0.375
He_2_	C_50_	−0.251	−0.135	−0.193	0.115	0.324
Ne_2_	C_50_	−0.252	−0.136	−0.194	0.116	0.324
Ar_2_	C_50_	−0.256	−0.140	−0.198	0.116	0.337
	C_60_	−0.280	−0.114	−0.197	0.166	0.234
He_2_	C_60_	−0.266	−0.100	−0.183	0.166	0.201
Ne_2_	C_60_	−0.265	−0.101	−0.183	0.165	0.203
Ar_2_	C_60_	−0.263	−0.105	−0.184	0.158	0.215
	C_70_	−0.275	−0.116	−0.195	0.158	0.241
He_2_	C_70_	−0.261	−0.102	−0.182	0.159	0.207
Ne_2_	C_70_	−0.261	−0.102	−0.182	0.159	0.207
Ar_2_	C_70_	−0.262	−0.104	−0.183	0.159	0.211

*Atomic units*.

**Figure 3:**
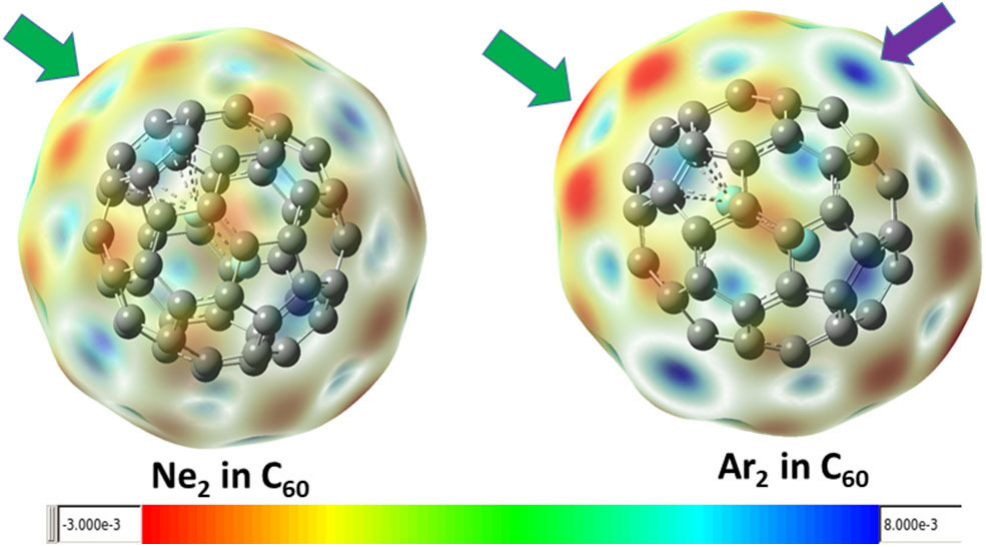
The mapped contour surface of the molecular electrostatic potential onto the van der Waals surface for Ne_2_ and Ar_2_ dimers encapsulated in C_60_ fullerene.

Figure 3 shows the contour surface of the molecular electrostatic potential mapped onto the van der Waals surface for Ne_2_ and Ar_2_ dimers encapsulated in C_60_ fullerene. It shows that the carbon atoms perpendicular to the Ng-Ng bond is more electronegative. This result is consistent with our Hirshfeld charge result, showing that Ng atoms become more positive and the neighboring carbon atoms in fullerene become more negative. Since Hirshfeld charges are reliable descriptors of electrophilicity and nucleophilicity, Figure 3 also shows that carbon atoms of the fullerene molecule become more nucleophilic, capable of donating more electrons to an electrophile.

**Figure 4:**
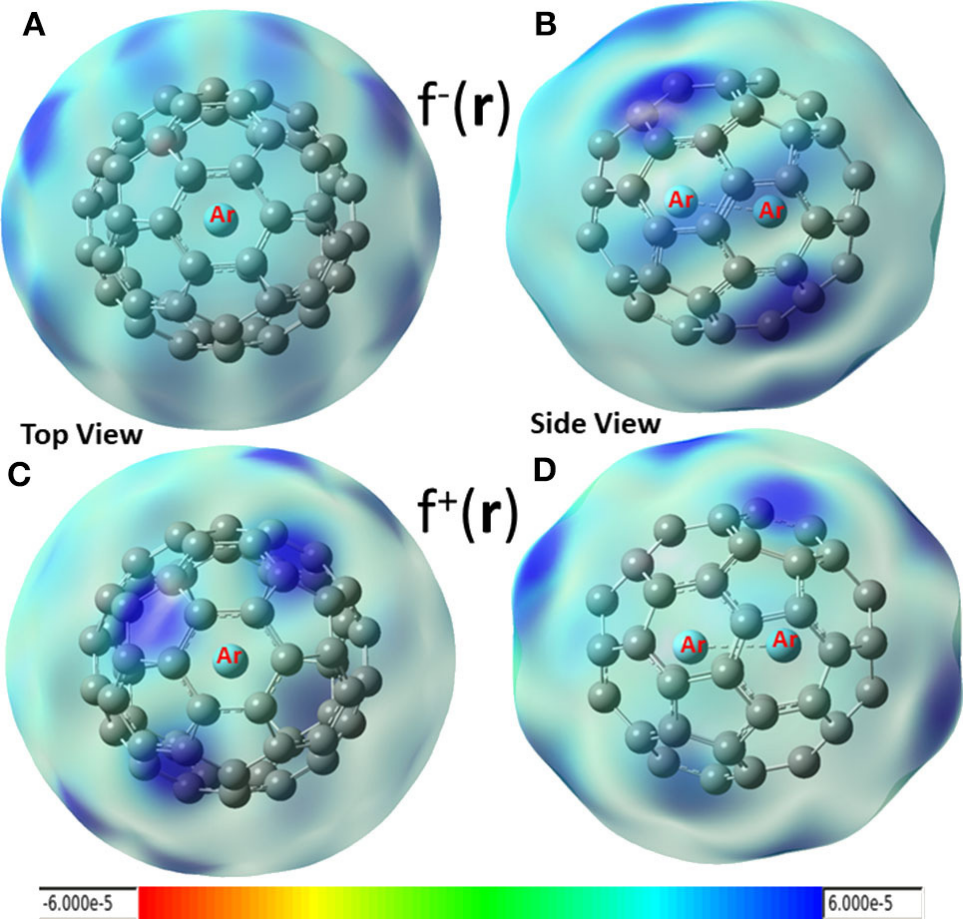
Electrophilic **(A,B)** and nucleophilic **(C,D)** Fukui functions for Ar_2_ dimer encapsulated in C_60_ fullerene.

In conceptual DFT, Fukui functions (Yang et al., 1984) can also be employed as local descriptors to predict nucleophilic and electrophilic attacks. Shown in Figure 4 are these local functions, where both electrophilic (Figures 4A,B) and nucleophilic (Figures 4C,D) Fukui functions for Ar_2_ dimer encapsulated in C_60_ fullerene are exhibited. From the figure, we found that (i) carbon atoms in fullerene can be both electrophilic (dark blue areas in Figures 4A,B) and nucleophilic (dark blue regions in Figures 4C,D), and (ii) nucleophilic regions in Figures 4C,D are drastically different from those of Figure 3 (red areas), suggesting that using Hirshfeld charge and Fukui charge, we obtain different results. According our recent findings, this discrepancy is not unusual. As our recent study demonstrated (Wang et al., 2019), results from the Hirshfeld charge should be more robust and reliable. Notice that Krapp and Frenking (2007) also observed that Ng_2_ molecules were oxidized inside fullerenes, implying that Ng_2_ became more electrophilic in return as electrons were transferred to C_60_, and this also made fullerenes more nucleophilic. This result was confirmed by Solà et al. (Osuna et al., 2009, 2011), also who unveiled that the combination of the LUMO stabilization, increased fullerene strain energy, and greater compression of the encapsulated Ng_2_ contributed to the reactivity change.

**Figure 5:**
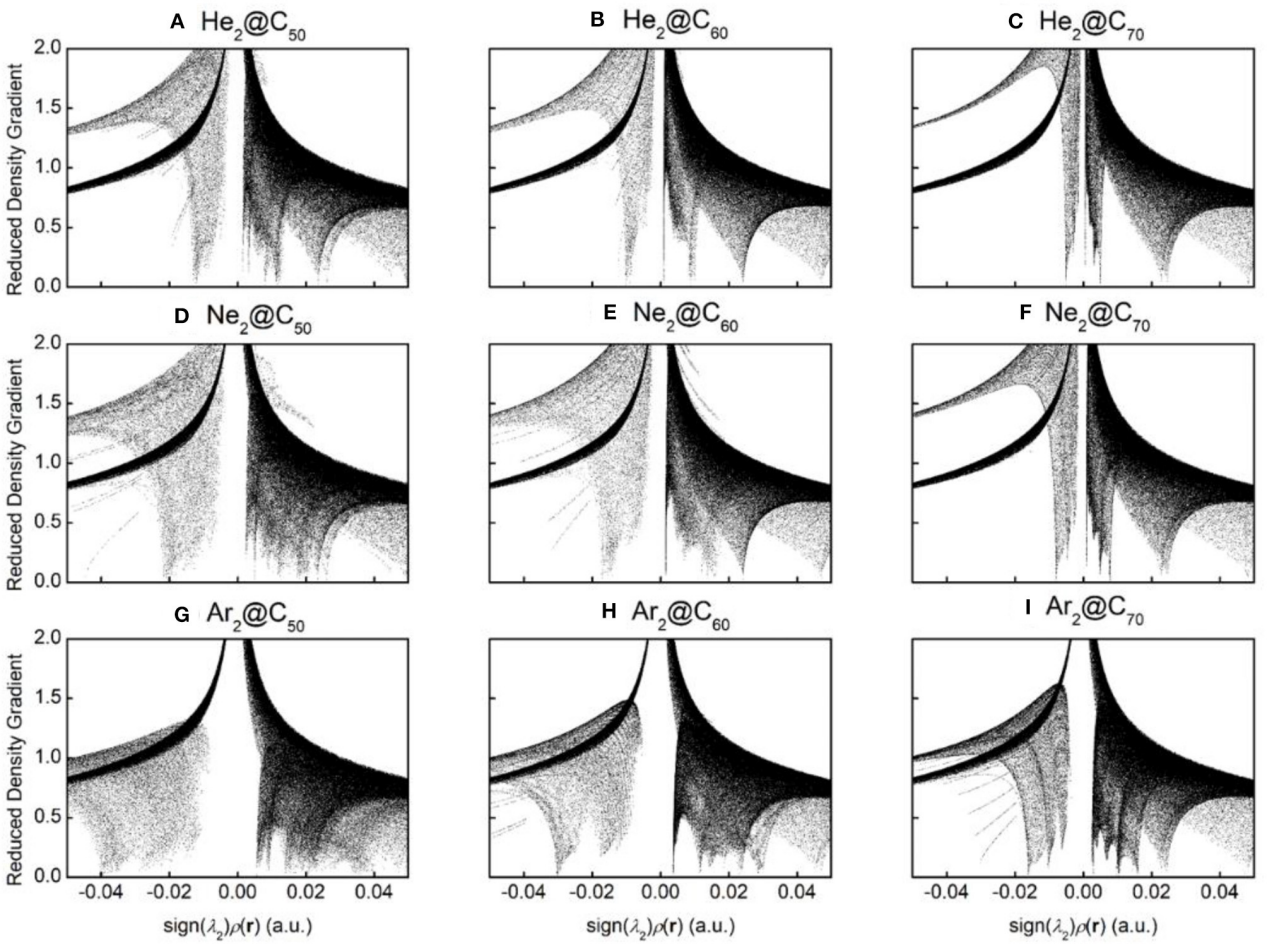
Diagrams of the reduced density gradient (RDG) vs. sign(λ_2_)ρ(r) for nine endohedral fullerenes: **(A)** He_2_@C50; **(B)** He_2_@C60; **(C)** He_2_@C70; **(D)** Ne_2_@C50; **(E)** Ne_2_@C60; **(F)** Ne_2_@C70; **(G)** Ar_2_@C50; **(H)** Ar_2_C60; and **(I)** Ar_2_@C70.

To qualitatively identify and distinguish weak interactions between Ng_2_ and fullerene molecules, noncovalent interaction (NCI) analysis (Johnson et al., 2010) was employed, whose results are shown in Figure 5. The reduced density gradient is large and positive in the regions far from the molecule, but it becomes small in the vicinity of both covalent and non-covalent interactions. To identify different types of interactions, the sign of Laplacian of the density is utilized and compared. Plotting low-gradient isosurfaces subject to a further low-density constraint enables real-space visualization of non-covalent interactions. Results in Figure 5 show that when He_2_ encapsulated from C_50_ to C_70_, a “spike” area becomes more apparent from −0.02 to 0 a.u., indicating that there existed more attractive interactions within the complexes as the fullerene cage becomes larger. The same trend is also observed for Ne_2_ and Ar_2_. These results are also in qualitative agreement with the total energy difference result as shown in Table 2.

Table 4 is the results from the two schemes (Parr and Yang, 1989; Liu, 2007) of the total energy partition for the nine Ng_2_-fullerene systems studied in this work. Different from the traditional binding energy or interaction energy partition, the total energy partition decomposes the total energy *E* of a molecular system in terms of a few components that are physiochemically meaningful. In DFT (Parr and Yang, 1989), we have E = T_S_ + E_xc_ + E_e_, where T_S_, E_xc_, and E_e_ are the non-interacting kinetic energy, exchange-correlation energy, and electrostatic interaction energy, respectively. An alternative scheme was proposed by one of us (Liu, 2007), where E = E_S_ + E_e_ + E_q_, where E_S_, E_e_, and E_q_ stand for the energetic contribution from three physiochemical effects, steric, electrostatic, and quantum (due to the exchange-correlation effects). There is a common component in these two partition schemes, the electrostatic energy E_e_. Considering the energy difference ΔE between two chemical processes sharing the same molecular fragments, we have ΔE = ΔT_S_ + ΔE_xc_ + ΔE_e_, and ΔE = ΔE_S_ + ΔE_e_ + ΔE_q_. Table 4 shows the values of these five components from the above two partition schemes using Ng_2_ and fullerene in vacuum as the reference. From the table, we found that in the first scheme (Parr and Yang, 1989; Liu, 2007), both ΔT_s_ and ΔE_xc_ contribute positively to ΔE <0, and it is the electrostatic contribution ΔE_e_ that contributes negatively to their stability, in order to make ΔE <0. Since E_e_ itself is negative in value (Liu, 2007), this result suggests that to put Ng_2_ dimer in the fullerene cage, one has to overcome the large electrostatic repulsion between Ng_2_ and fullerene. This result is further verified by the second total energy partition scheme (Liu, 2007), where we found that in this case both electrostatic and quantum (due to exchange-correlation effects) contribute negatively to ΔE <0 but the steric contribution ΔE_S_ is positive. This result shows that after Ng_2_ dimer is put into the cage, smaller space is occupied, but to do so, large electrostatic and Fermionic quantum repulsions have to be overcome. Notice that this total energy difference is different from the BSSE corrected interaction energy in Table 2 and our present results are consistent with what we observed elsewhere for other systems.

**Table 4:** Numerical results of the two total energy partition schemes using Ng_2_ and fullerene in vacuum as the references for the nine Ng_2_-fullerene systems studied in this work.

**Ng_**2**_**	**Fullerene**	**ΔE**	**ΔT_**s**_**	**ΔE_**xc**_**	**ΔE_**e**_**	**ΔE_**s**_**	**ΔE_**q**_**
He_2_	C_50_	0.44	−73.19	−11.90	85.57	−357.75	272.66
He_2_	C_60_	−3.66	−56.13	−7.91	60.37	−270.58	206.54
He_2_	C_70_	−7.33	−47.92	−5.54	46.09	−205.288	151.82
Ne_2_	C_50_	14.04	−184.03	−15.31	213.37	−709.51	510.17
Ne_2_	C_60_	−2.50	−125.76	−12.07	135.38	−531.19	393.36
Ne_2_	C_70_	−15.28	−62.23	−11.40	58.32	−379.22	305.59
Ar_2_	C_50_	202.08	−356.45	−11.79	570.33	−1018.14	649.90
Ar_2_	C_60_	97.83	−241.92	−27.79	367.51	−916.08	646.36
Ar_2_	C_70_	0.77	−142.95	−43.29	187.01	−838.78	652.54

*Units in kcal/mol*.

**Figure 6:**
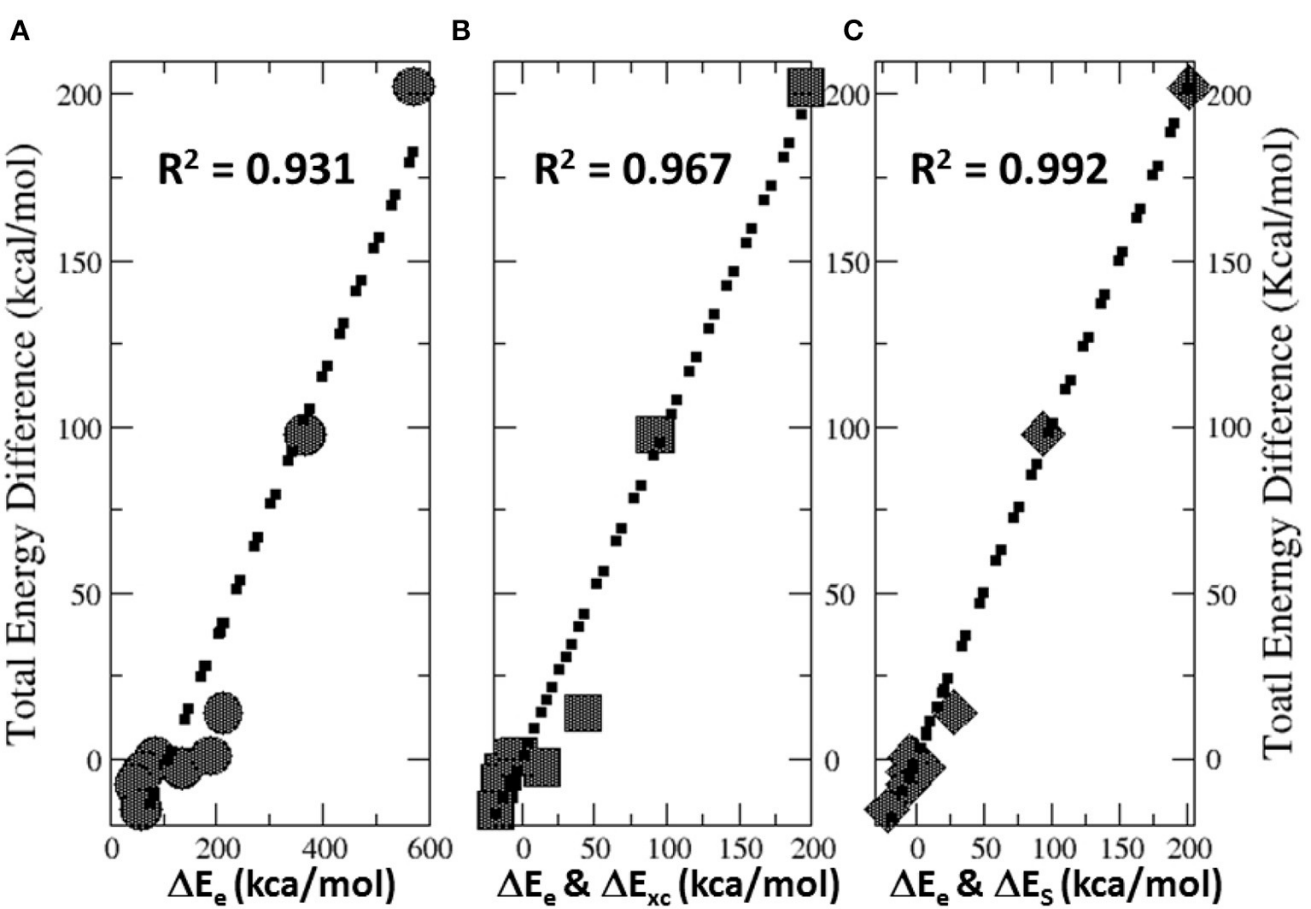
Strong linear correlations of the total energy difference ΔE with **(A)** the electrostatic energy difference ΔE_e_, **(B)** two-variable fitting with ΔE_e_ and ΔE_xc_, and **(C)** two-variable fitting with ΔE_e_ and ΔE_S_.

Figure 6 illustrates the total energy partition analysis to elucidate which energy component is dominant over others in contributing to the total energy difference. A strong linear correlation of the total energy difference ΔE with the electrostatic energy difference ΔE_e_ is shown in Figure 6A, suggesting that it is the electrostatic repulsion that dominates the energetic contribution, which agrees well with the numerical results in Table 4. If the strategy of two-variable fitting is employed, shown in Figures 6B,C, we found that ΔE_e_ combining with either ΔE_xc_ and ΔE_S_ yields even stronger correlations, indicating that contributions from the exchange-correlation and steric effects are minor yet indispensable.

To confirm the results in Tables 4, 5 shows the total bonding energy ΔE_bond_ analysis (Bickelhaupt and Baerends, 2000) results using the ADF package. In this analysis, ΔE_bond_ consists of contributions from the Pauli repulsion ΔE_Pauli_, steric interaction ΔE_steric_, electrostatic attraction ΔE_elstat_, orbital interactions ΔE_orb_, and dispersion Pauli repulsion ΔE_disp_, ΔE_bond_ = ΔE_Pauli_ + ΔE_steric_ + ΔE_orb_ + ΔE_disp_ + ΔE_elstat_. From the results in Table 5, it can be seen that the steric effect (even though its definition is substantially different) contributes favorably and the major opposite contribution is from the electrostatic interaction, agreeing well with our results in Table 4.

**Table 5:** The chemical bonding analysis results using the ADF package to analyze the bond energy between the Ng_2_dimer and fullerene fragments.

**Ng**	**Fullerene**	**ΔE_**Pauli**_**	**ΔE_**steric**_**	**ΔE_**orb**_**	**ΔE_**disp**_**	**ΔE_**elstat**_**	**ΔE_**bond**_**
He_2_	C_50_	29.65	16.71	−6.10	−5.99	−29.65	4.62
He_2_	C_60_	16.40	8.87	−4.30	−5.55	−16.40	−0.98
He_2_	C_70_	8.25	4.24	−3.03	−5.24	−8.25	−4.03
Ne_2_	C_50_	95.01	43.64	−7.62	−11.36	−95.01	24.66
Ne_2_	C_60_	50.91	21.36	−4.72	−10.98	−50.91	5.66
Ne_2_	C_70_	22.02	7.41	−2.94	−10.60	−22.02	−6.13
Ar_2_	C_50_	431.50	197.82	−31.52	−33.92	−431.49	132.39
Ar_2_	C_60_	244.49	103.07	−19.37	−34.79	−244.50	48.90
Ar_2_	C_70_	103.18	33.17	−11.53	−34.85	−103.18	−13.21

Units in kcal/mol.

*The energy decomposition analysis (EDA) in ADF dissects the interactions that constitute a chemical bond between fragments in a molecule. The total bonding energy ΔE_bond_ consists of contributions from the Pauli repulsion ΔE_Pauli_, steric interaction ΔE_steric_, electrostatic attraction ΔE_elstat_, orbital interactions ΔE_orb_, and dispersion Pauli repulsion ΔE_disp_, ΔE_bond_ = ΔE_Pauli_ + ΔE_steric_ + ΔE_orb_ + ΔE_disp_ + ΔE_elstat_*.

**Figure 7:**
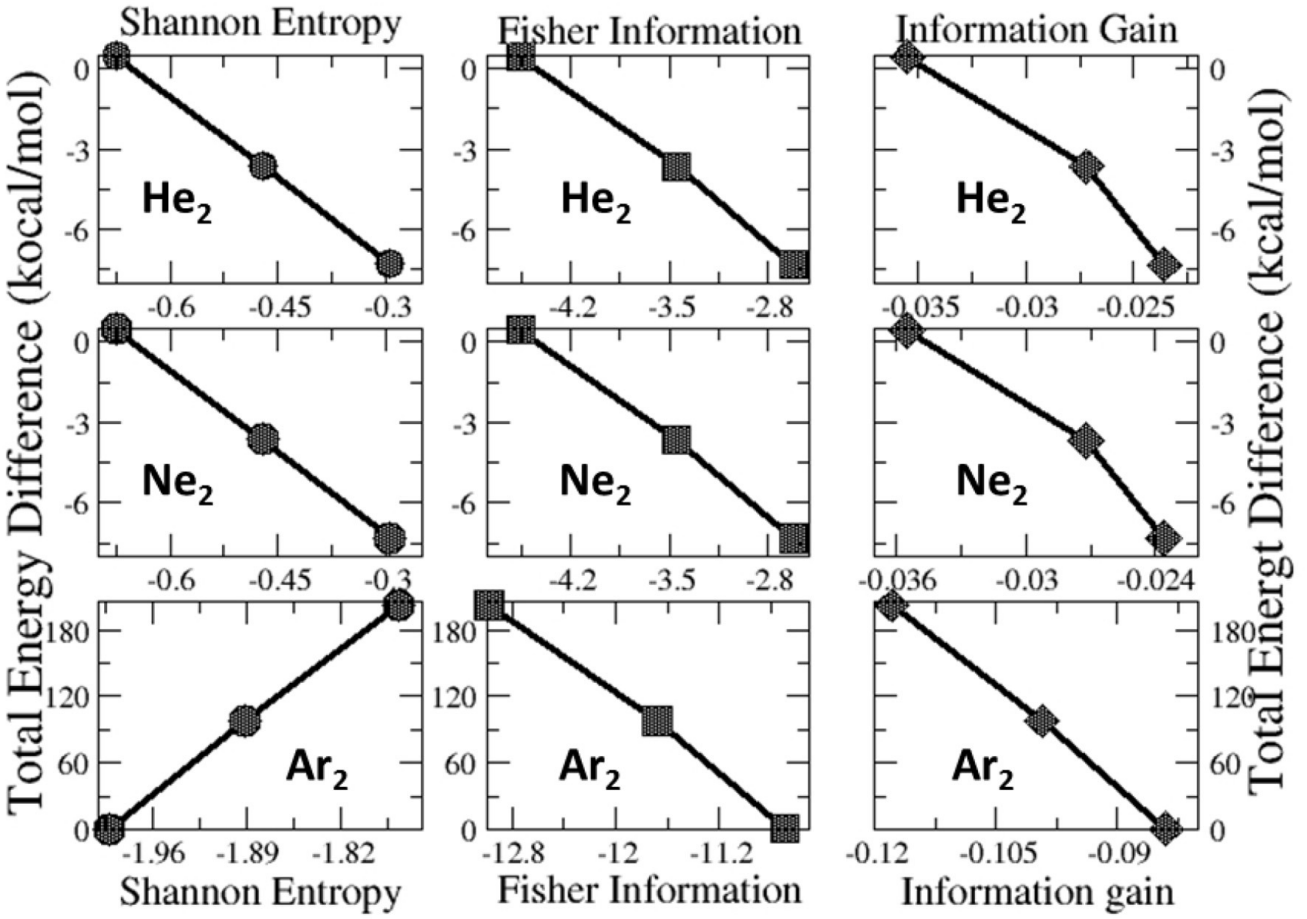
Strong linear relationships of the total energy difference ΔE with ITA quantities such as Shannon entropy, Fisher information, and information gain for He_2_, Ne_2_, and Ar_2_ encapsulated in C_50_, C_60_, and C_70_ fullerenes.

Lastly, we examine the results of eight quantities from the information-theoretic approach, which are shown in Table 6, including Shannon entropy (Shannon, 1948), Fisher information (Fisher, 1925), Ghosh-Berkowitz-Parr entropy (Ghosh et al., 1984), information gain (Kullback and Leibler, 1951), 2nd and 3rd orders of absolute Rényi and relative Rényi entropy (Rényi, 1970; Nagy and Romera, 2015). These quantities each have their own physio-chemical meaning and thus representing different yet intrinsic properties of molecular systems. For example, Shannon entropy is a measure of the electron density distribution uniformity, Fisher information gauges the heterogeneity of the same electron density distribution, and information gain is a robust measurement of regioselectivity, electrophilicity, and nucleophilicity, with its first-order approximation yielding the Hirshfeld charge (Liu et al., 2014). To make sense of these ITA results, Table 7 lists the correlation coefficient of these quantities with respect to either Ng_2_ or fullerene types, and Figure 7 displays illustrative example of strong linear relationships of the total energy difference ΔE with Shannon entropy, Fisher information, and information gain for He_2_, Ne_2_, and Ar_2_ encapsulated in three fullerenes. Strong correlations are seen in most cases, as shown in the figure, but as highlighted in Table 7, these strong correlations are true only for Ng_2_ dimers cross different fullerenes. If one fits a fullerene with three Ng_2_ dimers, no such strong correlation is often observed. Also, we tried to put all data points in one plot, and no significant correlation was observed either. Strong correlations in Figure 7 show that ITA quantities could be good descriptors of change trends for Ng_2_ dimers encapsulated in fullerene cages. For instance, for He_2_ encapsulated in from C_50_ to C_70_, binding energy increases, so does the Shannon entropy, due to the more delocalization of the electron density distribution. Another example is the information gain, which is a direct measure of the Hirshfeld charge. Again, for He_2_ dimer, from C_50_ to C_70_, the difference in information gain decreases, leading to the decrease in Hirshfeld charge, which is in good agreement with the result in Table 2. We noticed the abnormality of Shannon entropy for Ar_2_ dimer in Figure 7, whose trend is opposite to that of He_2_ and Ne_2_. The reason is unknown. More studies are in need.

**Table 6:** Numerical results of eight ITA quantities.

**Ng**	**Fullerene**	**ΔS_**s**_**	**ΔI_**F**_**	**ΔS_**GBP**_**	**ΔI_**G**_**	**ΔR_**2**_**	**ΔR_**3**_**	**Δ^*r*^R**_**2**_	**Δ^*r*^R**_**3**_
He_2_	C_50_	−0.674	−4.561	−0.658	−0.035	0.183	0.096	0.596	0.597
He_2_	C_60_	−0.470	−3.450	−0.509	−0.027	0.183	0.096	0.597	0.598
He_2_	C_70_	−0.293	−2.617	−0.390	−0.024	0.183	0.096	0.598	0.598
Ne_2_	C_50_	−0.773	−9.045	−1.003	−0.067	2.448	2.227	1.274	1.275
Ne_2_	C_60_	−0.596	−6.772	−0.774	−0.053	2.461	2.246	1.278	1.279
Ne_2_	C_70_	−0.456	−4.835	−0.573	−0.043	2.470	2.261	1.281	1.282
Ar_2_	C_50_	−1.777	−12.980	−1.943	−0.118	2.979	2.370	1.508	1.510
Ar_2_	C_60_	−1.891	−11.679	−1.864	−0.099	3.025	2.408	1.516	1.517
Ar_2_	C_70_	−1.991	−10.694	−1.848	−0.084	3.062	2.441	1.521	1.523

*Atomic units*.

**Table 7:** Correlation coefficients *R*^2^ values between ΔE and eight ITA quantities for three Ng_2_ dimers with different fullerene systems (first three rows) and for three fullerenes with different Ng_2_ dimers (last three rows).

***R*^**2**^**	**ΔS_**s**_**	**ΔI_**F**_**	**ΔS_**GBP**_**	**ΔI_**G**_**	**ΔR_**2**_**	**ΔR_**3**_**	**Δ^*r*^R**_**2**_	**Δ^*r*^**R**_**3**_**
He_2_	1.000	0.998	0.999	0.964	0.999	0.874	0.996	0.997
Ne_2_	1.000	0.999	0.999	0.999	1.000	1.000	1.000	1.000
Ar_2_	0.998	0.997	0.890	0.999	0.998	0.999	0.995	0.995
C_50_	1.000	0.769	0.960	0.896	0.479	0.357	0.549	0.549
C_60_	0.995	0.846	0.970	0.888	0.437	0.316	0.500	0.501
C_70_	0.676	0.498	0.649	0.443	0.040	0.006	0.065	0.066

## Conclusions

To summarize, in this work, we investigated the possibility of making noble gas reactive through the means of fullerene encapsulation. To that end, we built a total of nine model systems, with three Ng_2_ dimers (He_2_, Ne_2_, and Ar_2_) encapsulated in three fullerene (C_50_, C_60_, and C_70_) cages. Quantum molecular dynamics simulations were employed to explore the potential energy surface of Ng_2_ inside the fullerene molecule. To obtain the lowest energy structure it was further examined through a few well-established analytical tools such as conceptual density functional theory, information-theoretic approach, total energy decomposition, bonding energy decomposition, and others. Our results show that bond distances of Ng_2_ inside fullerene become substantially smaller and noble gas atoms become more electrophilic. In return, these noble gas dimers make fullerene molecules more nucleophilic. Using these analytical tools, we appreciate the nature and origin of these structure and reactivity changes. Our results and conclusions drawn from the present study should provide more understanding and new insights from the viewpoint of changing the perspectives of noble gas reactivity.

## Data Availability Statement

The raw data supporting the conclusions of this article will be made available by the authors, without undue reservation, to any qualified researcher.

## Author Contributions

ML performed the calculations and analyses and prepared the draft. XH and BW helped in calculations and analyses. DZ and CR managed progression of the project. PC initiated the project and revised the draft. SL worked on the plan, assisted the calculations and analyses, and revised the manuscript. All authors contributed to the article and approved the submitted version.

## Conflict of Interest

The authors declare that the research was conducted in the absence of any commercial or financial relationships that could be construed as a potential conflict of interest.
